# Effects of Synbiotics on Inflammatory Markers in Patients With Type 2 Diabetes Mellitus

**DOI:** 10.5539/gjhs.v7n7p1

**Published:** 2015-03-26

**Authors:** A. Akram Kooshki, Tahereh Tofighiyan, Mohamad Hassan Rakhshani

**Affiliations:** 1 School of Medicine, Sabzevar University of Medical Sciences, Sabzevar, Iran; 2 School of Nursing, Sabzevar University of Medical Sciences, Sabzevar, Iran; 3 School of Health Sciences, Sabzevar University of Medical Sciences, Sabzevar, Iran

**Keywords:** symbiotic, inflammation, type 2 diabetes mellitus

## Abstract

**Introduction:**

With regard to the relationship between inflammation and insulin resistance and due to the lack of researches conducted about the effect of synbiotics on inflammatory markers in diabetes patients, this study was designed to investigate the effect of these markers.

**Methods:**

A double-blind, placebo-controlled trial was conducted among 44 type 2 diabetes patients. They were randomized to symbiotic or placebo group. Patients in the symbiotic group received one symbiotic tablet daily for 8 weeks whereas the placebo group received 1 placebo tablet. The hs-CRP concentration, TNF-α and IL-6 were measured by using ELISA kits. The dietary intakes of patients were assessed at the first and the end of the study and analyzed by Nutritionist IV. Data were analyzed by using SPSS 16.0 via paired and independent t- test.

**Results:**

Anthropometric and dietary data were not significantly different between the two groups at the first and the end of the study. The serum concentrations of hs-CRP, IL-6 and TNF-α decreased significantly in the symbiotic group at the end of week 8 compared to baseline (p<0.05). Also, no significant changes were seen in the placebo group (p>0.05). The reduction in inflammatory markers in the symbiotic group were significant in compared to the placebo group (P<0.05).

**Conclusions:**

Symbiotic supplementation can reduce serum hs-CRP, IL-6 and TNF-α concentrations, a risk factor for cardiovascular diseases.

## 1. Introduction

Diabetes mellitus type 2 is an impendent risk factor for morbidity and mortality of cardiovascular diseases. Relative risk of cardiovascular disease is 2-3 times high in diabetic men and 3-4 times in diabetic women comparison to non-diabetic controls (Barrett et al., 1991; Steven et al., 1998; William et al., 1979). Risk factors for cardiovascular diseases such as lipid abnormalities and hypertensions are prevalent in diabetic patients but they cannot explain the high frequency of cardiovascular disease in these patients (Stenvinkel et al., 2004). Some studies have indicated that high concentrations of serum inflammation markers are an important risk factor for cardiovascular disease in type’s diabetes patients (Reaven et al., 1988; Defronzo et al., 1991; Despres et al., 1996). Few reports are available on the correlation between hs-C-reactive protein (hs-CRP) and cardiovascular disease in type 2 diabetes patnts (Japer et al., 1999; Stehouwer et al., 2002). Also, tumor necrosis factor-α (TNF-α) is an inflammatory cytokine that can alter in sensitivity in different ways by attenuating insulin receptor Signaling pathways, by decreasing glucose transporter – 4 in adiposities and by suppressing adiponectin (Calle et al., 2012). Also, TNF-α increases the expression of genes encoding IL-6 and Mcp-1 and contributes to the progression of atherosclerosis (Calle et al., 2012).

Interleukin- 6 (IL-6) is inflammatory markers that the main its effects is the induction of hepatic CRP production which to be an independent, major risk factor for cardiovascular disease (Ridker et al., 2003). Recent studies have proposed that IL-6 could be involved in insulin resistance and diabetes and its complications (Bastard et al., 2006; Ray et al., 2009).

Some studies were done to find treatment ways for lowing inflammation in diabetes patients (Alokail et al., 2013). Although, limited studies have been done on the effects of probiotic and synbiotic supplementation on serum systemic inflammation markers such as CRP, IL-6 and TNF-α in patients with type2 diabetes (Kootte et al., 2012; Musso et al., 2011). The finding of these studies have been contradictory.

## 2. Methods

### 2.1 Data Source and Study Population

This study was a randomized double-blind, placebo- controlled trial. Forty-four type 2 diabetic patients (16 men and 28 women) were recruited from diabetic patients of diabetes clinic of Vaseei Hospital in Sabzevar, Iran. Type 2 diabetes was diagnosed according to the criteria of world health organization (World Health organization., 1985). The patients in this study did not have inflammatory and infectious diseases including hepatitis, and none of them received probiotic and prebiotic and omega-3 fatty acids and L-carnitine supplement and steroidal and non-steroidal anti-inflammatory drugs. Participating were received metformin, glybenglamide and insuline for controlling blood sugar. During this study, the diabetic patients, type and dose of drugs did not altered for any of the patients.

Patients were randomized to symbiotic (pre and probiotic) or placebo group subjects in the symbiotic group received one symbiotic tablet daily for 8 weeks and the placebo group received 1 placebo tablet.

Patients were advised no change in their dietary habits, physical activities and drug regiments. At the first and the end of study, 5ml blood was collected from each patient after a 12- to 14-h fast. Blood samples after clotting at room temperature (20-25 °C) were centrifuged at 2000 rpm for 10 min. The sera were separated into small aliquots and frozen at -70 °C until they were used.

The serum hs-CRP concentration was determined by using enzyme-like immune-sorbent assay (ELISA) kits (Monobind, Inc., Lake Forest, Calif., USA). The CV for serum CRP was 2.9%. Serum TNF-α and IL-6 were measured by using ELISA kits (Diaclone, Beancon, France). The CVs for serum TNF-α and IL-6 were 5.6% and 5.1% respectively.

Patients were weighted and heighted at baseline and at the end of week 8. In addition, the dietary intakes of patients were assessed using a 2-day food recall at the first and at the end of study. Subject’s diets were analyzed by Nutritionist IV software (N squared computing, san Bruno, Calif., USA).

To ascertainment of subjects’ compliance, we provided each patient with a fixed number of tablets and instructions to return the unconsumed tablets at the end of the study. The compliance rate for each patient was determined according to the number of returned tablets. The compliance of all subjects was more than 90% and no adverse events were reported.

### 2.2 Statistical Analysis

Statistical analysis of the data was performed by using statistical package for the social sciences (SPSS, Inc., Chicago, III., USA) for windows version 15. As all quantitative parameters were normally distributed according to the Kolmogorov-Smirnov test, we used the t-test and the paired t-test to compare parameters between and within groups, respectively. The differences were considered significant at P<0.05.

### 2.3 Ethnics

The study protocol was approved by the Ethics committee of Sabzevar University of Medical Sciences. This study was in adherence with the Declaration of Helsinki Written informed consent which was obtained from all patients.

## 3. Results

The patients’ baseline characteristics did not differ significantly between the two groups (Table 1).

**Table 1:** Baseline characteristics and anthropometric data of diabetic patients in the symbiotic and placebo groups

Characterisfics	Symbiotic(n=22)	Placebo(n=22)
**Age (years)**	53.45± 10.8	54.5 ± 11.10
**Duration (months)**	7.25± 5.2	7.45± 5.4
**Sex**		
**Men, n**	8(36.36%)	8(36.36%)
**Women, n**	14(63.63%)	14(63.63%)
**Smoker, n**	0	0
**BMI, Kg/m^2^**	22.79±2.7	22.47± 2.38

Anthropometric and dietary data were not statistically different between the two groups at the baseline and the end of the study. In addition, these factors did not significantly change within each group during the study.

The serum concentrations of hs-CRP, IL-6 and TNF-α decreased significantly in the symbiotic group at the end of the study compared to the first study (p<0.05). Also, no significant changes were observed in the placebo group (p>0.05). The reduction in serum concentrations of hs-CRP, IL-6 and TNF-α in the symbiotic group were significant in compared to the placebo group (P<0.05, Table 2).

**Table 2:** serum concentrations of hs-CRP, IL-6 and TNF- α in the symbiotic and placebo groups (n=22 for all values)

Serum parameter and groups	Baseline	Week8	P-value
**hs-CRP, mg/L**			
**Synbiotic**	4.94± 2.36	4.15± 1.96	0.0001
**Placebo**	5.00± 2.31	5.08± 2.27	0.412
**IL- 6, ng/L**			
**Synbiotic**	9.19± 5.97	8.12± 5.02	0.011
**Placebo**	8.89± 5.18	8.86± 5.03	0.489
**TNF-α, ng/L**			
**Sybiotic**	8.03± 2.73	7.36± 2.61	0.002
**Placebo**	8.64± 2.61	8.65± 2.57	0.956

## 4. Discussion

Systemic inflammation is a common in the diabetes patients (Despres et al., 1996). It may lead to various complications including the cardiovascular disease in diabetes patients. Also, no treatment strategy for reducing inflammation in diabetes patients has been established yet (Calle et al., 2012).

In the present study, symbiotic supplementation significantly decreased serum hs-CRP, IL-6 and TNF-α. There is disagreement on the effect of synbiotic on serum systematic inflammation marker in diabetic patients.

Evidence is available on the anti inflammatory properties of probiotics. Some researches showed beneficial of probiotic using in diabetes type 2 but (Alokail et al., 2013) others reported no effect (Diamant et al., 2011; Esteve et al., 2011). Hatakka et al. showed that Lactobacillus rhamnosus GG increased serum IL-1B and no effect on IL-6 and TNF-α and IL-10 (Hatakka et al., 2003).

In contrast, Marschan and et al showed that infants receiving probiotic had higher plasma levels of CRP and IL-10 comparison to group placebo (Marschan et al., 2008).

Asemi et al., showed that daily administration of multispecies probiotic supplements decreased in serum hs-CRP in diabetes patient in 8 weeks but in their study, effects of probiotc supplements was not evaluated on serum IL-6 and TNF-α (Asemi et al.,2013).

CRP and IL-6, the two most sensitive markers of inflammation have been elevated in patients with type 2 diabetes (Pickup et al., 1997).

Also, high CRP level is reported to be a risk factor for progression type 2 diabetes (Voulgari et al., 2006).

Mazloom and et al indicated that administration of 1500 mg probiotic capsules twice daily to 20 patients with type2 diabets for 6 weeks reduced serum IL-6 and elevated serum hs-CRP levels although not statistically significant(Mazloom et al.,2013).

## 5. Conclusion

In conclusion, symbiotic supplementation can reduce serum hs-CRP, IL-6 and TNF-α concentrations, a risk factor for cardiovascular diseases.
